# Association of Diabetes with Oral Cancer- an Enigmatic Correlation

**DOI:** 10.31557/APJCP.2020.21.3.809

**Published:** 2020-03

**Authors:** Mounika Reddy Mekala, Balaji Babu Bangi, Jayalatha N, Rajasekhar Reddy Lebaka, Lakshmi Kavitha Nadendla, Uday Ginjupally

**Affiliations:** 1 *Department of Oral Medicine and Radiology, Kamineni Institute of Dental Sciences, Narketpally, Telangana, *; 2 *Department of Radiology,*; 3 *Department of Microbiology, MNJ institute of oncology and regional cancer center, Hyderabad, India. *

**Keywords:** Alcohol, chewing tobacco, Diabetes Mellitus (DM), Metformin, oral cancer and tobacco

## Abstract

**Introduction:**

Association of diabetes mellitus (DM) with head and neck cancers (HNC) is still controversial. In some studies, diabetic patients had an increased risk of cancer at some HNC subsites like oral cancer, while in other studies this risk was decreased. So, the present study aims to evaluate the association of diabetes mellitus, oral cancer with and without metformin and the role of habits in association with DM and metformin in the etiology of oral cancer.

**Materials and methods:**

This study was undertaken in the Kamineni Institute of Dental Sciences in collaboration with MNJ Institute of Oncology and Regional Cancer Centre, Hyderabad. The study includes 2 main groups, they are 500 Oral cancer patients and Control group includes 500 age and gender-matched patients with habits without any oral precancerous lesion/conditions. Odds ratios (OR) and 95% confidence intervals (CI) were estimated using unconditional logistic regression.

**Results:**

Out of 1000 subjects inverse relation of DM with oral cancer was observed. On comparison between oral cancer, diabetes and habits in study group and control group, decreased risk was observed with smokers (OR: 1.131and 95%CI: 0.68 -1.86) and non-chewers (OR: 2.43 and 95% CI: 1.31 - 4.49) and non-alcoholics (OR: 1.78 and 95% C.I:1.18 - 2.68). Metformin use among diabetic participants was associated with a decreased risk of oral cancer (OR: 0.51 and 95% C.I: 0.33 - 0.77). A negative association was observed in smokers (OR: 0.19 and 95% C.I.: 0.078 - 0.459), non-chewers (OR: 0.24 and 95% C.I : 0.11- 0.53) and non-alcoholics (OR: 0.46 and 95% C.I. : 0.29 - 0.727).

**Conclusion:**

Thus the present population based study results suggest an inverse association of DM and oral cancer with metformin and negative association of habits with DM and Metformin in etiology of oral cancer.

## Introduction

Cancer is the 2^nd^ leading cause of death worldwide, accounting for approximately an estimated 9.6 million deaths in 2018 (WHO, 2018). Oral cancer which is a subtype of head and neck cancer is any cancerous tissue growth located in the oral cavity (Khan, 2012). Oral cancer accounts for approximately 4-5% of all cancers and is the sixth most common cancer. While tobacco, alcohol use and infection with oncogenic Human papillomavirus (HPV) are estimated risk factors for head and neck cancer (HNC) emerging evidence suggests that abnormalities of glucose metabolism and diabetes may also play a role (Rivera, 2015). Therefore if diabetes is associated with an increase in the risk of cancer, this may have a tremendous effect on health worldwide (Boccia et al., 2012).

The association between diabetes mellitus (DM) and the increased risk of certain cancers, such as liver, pancreatic, colon, kidney, bladder, endometrial, and breast cancer is well established, while the risk of prostate cancer is decreased among diabetic patients. Although some studies with DM have also been associated with HNC, these results are still controversial (Gonsalves et al., 2015). In some studies, diabetic patients had an increased risk of cancer at some HNC subsites like oral cancer, while in other studies this risk was decreased. One possible explanation for the inverse association between DM and some kinds of cancers is metformin use among diabetic patients (Carvalho et al., 2016). Recently, increasing evidence has been presented for the potential anti-cancer and anti-metastasis effect of metformin (Bashirelahi et al., 2015). This the first study of its kind to study the association of diabetes and metformin with oral cancer patients with effects of habits. 

*Aim and Objectives*

This study aims to evaluate the association between DM and Oral cancer, as well as the role of metformin use with risk of Oral cancer. Other objectives of the study include comparison of DM in oral cancer patients and control patients with habits, comparison of metformin users in oral cancer and controls and to assess the protective role of metformin in controls with habits. 

## Materials and Methods

The present study was undertaken in the Department of Oral Medicine, Diagnosis and Radiology, Kamineni Institute of Dental Sciences in collaboration with MNJ Institute of Oncology and Regional Cancer Centre, Hyderabad. The study includes 2 main groups, study group of 500 Oral cancer patients (Clinically and histopathologically diagnosed Oral cancer patients) who were referred to MNJ cancer institute from regional hospitals of Telangana and control group includes 500 age and gender-matched patients with habits and without any oral precancerous lesion/conditions reported to Department of Oral Medicine and radiology. 

Informed consent of the patient was taken for the study. Institutional ethical clearance and MNJ cancer institute ethical clearance has been taken. Inclusion criteria included patients of age group above 15 years of both genders with habits and diagnosed as oral cancer. The control group includes age and gender-matched patients with habits without any potentially malignant diseases (PMD). Patients below 15 years of age, patients with any other systemic conditions and on any medication affecting blood glucose levels and patients having any PMD’s were excluded.

Participants were interviewed using a standardized questionnaire to collect information about age, sex, DM and medication, tobacco consumption, and alcohol consumption are recorded. Blood samples are collected from cases and controls and fasting blood glucose levels and 2hr postprandial levels are estimated. Glucose oxidase - peroxidase GOD/POD method is used for the estimation of glucose levels. It is based on Trinder’s method.

According to the American Diabetes Association (ADA) 2018 Guidelines it was considered positive for diabetes when values were 

FPG ≥126 mg/dL (7.0mmol/L)* (Fasting is defined as no caloric intake for ≥8 hours)

PG ≥200 mg/dL (11.1mmol/L) during OGTT (75g)* Using a glucose load containing the equivalent of 75g anhydrous glucose dissolved in water.

*Statistical Analysis*

All the findings were entered in Microsoft Excel using SPSS 20.0 software and Odds ratios (OR) and 95% CI were estimated using unconditional logistic regression. The degree of freedom between variables was also observed. P-value < 0.05 represents statistically significant and P-value > 0.05 represents statistically non-significant.

## Results

A total of 1,000 patients were assessed as eligible to participate in the study. Out of 1,000, 500 patients were diagnosed with Oral cancer and 500 controls, age and gender-matched patients with habits without any oral precancerous lesion/conditions were included. Among 500 cases and 500 controls, male predominance was seen of 79.8% and 87.0% respectively. There were a total of 133 participants with DM, 54 (10.8%) cases and 79 (15.8%) controls (Table 1). Oral cancer is again divided into 10 subsites with the highest percentage on the tongue (31.8%) (Table1). Out of 1,000 subject’s inverse relation of DM with oral cancer was observed. But the risk reduction was statistically significant when all individuals (OR: 1.55 and 95% CI: 1.07 - 2.24) are compared and among the males (OR: 1.60; and 95%CI: 1.04 - 2.46) (Table 2).

On comparison of the relation between DM with oral cancer in different sites of oral cancer, no statistical significance was observed (Table 3). On comparison between oral cancer, diabetes and habits in study group and control group, decreased risk was observed with smokers (OR: 1.131and 95%CI: 0.68 -1.86) and non-chewers (OR: 2.43 and 95% CI: 1.31 - 4.49) and non-alcoholics (OR: 1.78 and 95% C.I:1.18 - 2.68) (Table 4).

Metformin use among diabetic participants was associated with a decreased risk of oral cancer (OR: 0.51 and 95% C.I: 0.33 - 0.77). A negative association was observed in males (OR: 0.48 and 95% C.I: 0.30- 0.78) (Table 5). A negative association was observed in smokers (OR: 0.19 and 95% C.I.: 0.078 - 0.459), non-chewers (OR: 0.24 and 95% C.I : 0.11- 0.53) and non-alcoholics(OR: 0.46 and 95% C.I. : 0.29 - 0.727) (Table 6). 

**Table 1:** Selected Characteristics of the Study Sample by Case/control Status

		Cases (N=500)		Controls (N=500)	
		N	%	N	%
Sex	Male	399	79.8	435	87.0
	Female	101	20.2	65	13.0
Smoking Status	Smokers	147	29.4	229	45.8
	Non Smokers	353	70.6	271	54.2
Tobacco Chewing	Chewers	312	62.4	230	46.0
	Non Chewers	188	37.6	270	54.0
Alcohol Status	Alcoholic	106	21.2	110	22.0
	Non Alcoholic	394	78.8	390	78.0
Presence Of DM	Diabetic	54	10.8	79	15.8
	Non Diabetic	446	89.2	421	84.2
Site Of Oral Cancer	Tongue	159	31.8		
	LBM	128	25.6		
	RBM	64	12.8		
	Alveolus	24	4.8		
	Fom	28	5.6		
	Hard Palate	39	7.8		
	Soft Palate	20	4.0		
	GBS	13	2.6		
	Lip	8	1.6		
	Rmt	17	3.4		

N, Number of patients; DM, Diabetes Mellitus; LBM, Left buccal mucosa; RBM, Right buccal mucosa; FOM, Floor of the mouth; GBS, Gingivobuccal sulcus; RMT, Retro molar trigone.

**Table 2:** Odds Ratios (OR) and Confidence Intervals (CI) of the Association between Diabetes Mellitus (DM) and Oral Cancer for all Participants and Stratified by Sex

		Group		P-Value	Odds Ratio	95% C.I.For Odds Ratio	
		Study Group (%)	Control Group (%)			Lower	Upper
All Individuals	Non-Diabetic	446 (51.4)	421 (48.6)	0.021*	1.550	1.070	2.246
	Diabetic	54 (40.6)	79 (59.4)				
Female	Non Diabetic	85 (63.4)	49 (36.6)	0.165	1.735	.798	3.773
	Diabetic	16 (50.0)	16 (50.0)				
Male	Non Diabetic	361 (49.2)	372 (50.8)	0.029*	1.609	1.049	2.468
	Diabetic	38 (37.6)	63 (62.4)				

**Table 3:** Odds Ratios (OR) and Confidence Intervals (CI) of the Association between Diabetes Mellitus (DM) and Oral Cancer Stratified by Site

Site	Non - Diabetic (%)	Diabetic (%)	P-Value	Odds Ratio	95% C.I	
					Upper	Lower
Tongue	142 (89.3)	17 (10.7)	0.457	0.756	0.362	1.579
LBM	113 (88.3)	15 (11.7)	0.076	1.938	0.934	4.02
RBM	60 (93.8)	4 (6.3)	0.150	2.076	0.769	5.60
Alveolus	22 (91.7)	2 (8.3)	0.234	2.895	0.503	16.67
FOM	21 (75.0)	7 (25)	0.240	2.826	0.499	15.99
Hard Palate	35 (89.7)	4 (10.3)	0.296	2.182	0.505	9.43
Soft Palate	20 (100.0)	0 (0.0)	0.314	3.353	0.318	35.36
GBS	9 (69.2)	4 (30.8)	0.54	.458	0.036	5.78
RMT	8 (100)	0 (0)	1.000	1.000	0.10	9.61
Lip	16 (94.1)	1 (5.9)	.276	2.275	0.51	9.98

LBM, Left buccal mucosa; RBM, Right buccal mucosa; FOM, Floor of the mouth; GBS, Gingivobuccal sulcus; RMT, Retro molar trigone.

**Table 4:** Odds Ratios (OR) and Confidence Intervals (CI) of Oral Cancer According to Diabetes Mellitus (DM) by Smoking, Chewing Tobacco and Alcohol

		Group		P-Value	Odds Ratio	95% C.I. For Odds Ratio	
		Study Group (%)	Control Group (%)			Lower	Upper
Non Smokers	Non Diabetic	309 (56.4)	239 (43.6)	0.804	0.940	0.579	1.528
	Diabetic	44 (57.9)	32 (42.1)				
Smokers	Non-Diabetic	137 (42.9)	182 (57.1)	0.001*	3.538	1.726	7.251
	Diabetic	10 (17.5)	47 (82.5)				
Non Chewers	Non Diabetic	173 (43.7)	223 (56.3)	0.005	2.431	1.315	4.493
	Diabetic	15 (24.2)	47 (75.8)				
Chewers	Non Diabetic	273 (58.0)	198 (42.0)	0.630	1.131	0.685	1.869
	Diabetic	39 (54.9)	32 (45.1)				
Non Alcoholic	Non Diabetic	351 (52.3)	320 (47.7)	0.005	1.786	1.186	2.688
	Diabetic	43 (38.1)	70 (61.9)				
Alcoholic	Non Diabetic	95 (48.5)	101 (51.5)	0.579	0.770	0.305	1.940
	Diabetic	11 (55.0)	9 (45.0)				

**Table 5:** Odds Ratios (OR) and Confidence Intervals (CI) of Oral Cancer According to Diabetes Mellitus (DM) and Metformin Use for all Participants and Stratified by Sex

		Group		P-Value	Odds Ratio	95% Ci	
		Study Group (%)	Control Group			Upper	Lower
All Individuals	Non Diabetic	446 (89.2)	421 (84.2)		1		
	Diabetic With Metformin Use	39 (7.8)	72 (14.4)	0.001*	0.511	0.339	0.772
	Diabetic Without Metformin Use	15 (3.0)	7 (1.4)	0.128	2.02	0.817	5.010
Female	Non Diabetic	85 (84.2)	49 (75.4)		1		
	Diabetic With Metformin Use	12 (11.9)	15 (23.1)	0.070	0.461	0.200	1.065
	Diabetic Without Metformin Use	4 (4.0)	1 (1.5)	0.461	2.30	0.251	21.217
Male	Non Diabetic	361 (90.5)	372 (85.5)		1		
	Diabetic With Metformin Use	27 (6.8)	57 (13.1)	0.003*	0.488	0.302	.789
	Diabetic Without Metformin Use	11 (2.8)	6 (1.4)	0.215	1.88	0.691	5.162

**Table 6:** Odds Ratios (OR) and Confidence Intervals (CI) of Oral Cancer According to Diabetes Mellitus (DM) and Metformin Use by Smoking, Chewing Tobacco and Alcohol

		Group		P -value	Odds ratio	95% confidence interval for odds ratio	
		Study group (%)	Control group (%)			Upper	Lower
Non smoker	Non diabetic	446 (89.2)	421 (84.2)		1		
	Diabetic with metformin	39 (7.8)	72(14.4)	0.544	0.851	0.505	1.435
	Diabetic without metformin	15 (3.0)	7 (1.4)	0.061	4.254	0.934	19.374
Smoker	Non diabetic	137 (93.2)	182 (79.5)		1		
	Diabetic with metformin	6 (4.1)	42 (18.3)	0.000*	0.190	0.078	0.459
	Diabetic without metformin	4 (2.7)	5 (2.2)	0.929	1.063	0.280	4.032
Non chewer	Non diabetic	173 (92.0)	223 (82.6)		1		
	Diabetic with metformin	8 (4.3)	42 (15.6)	0.000*	0.246	0.112	0.537
	Diabetic without metformin	7 (3.7)	5 (1.9)	0.320	1.805	0.563	5.784
	Non diabetic	273 (87.5)	198 (86.1)		1		
Chewer	Diabetic with metformin	31 (9.9)	30 (13.0)	.290	.749	.439	1.279
	Diabetic without metformin	8 (2.6)	2 (0.9)	0.181	2.901	0.609	13.809
Non alcoholic	Non diabetic	351 (89.1)	320 (82.1)		1		
	Diabetic with metformin	32 (8.1)	63 (16.2)	0.001*	0.463	0.295	0.727
	Diabetic without metformin	11 (2.8)	7 (1.8)	0.463	1.433	0.549	3.740
Alcoholic	Non diabetic	95 (89.6)	101 (91.8)		1		
	Diabetic with metformin	7 (6.6)	9 (8.2)	0.717	0.827	0.296	2.309
	Diabetic without metformin	4 (3.8)	0 (0.0)	0.121	2.801	0.409	12.309

## Discussion

500 oral cancer patients included in the study group were all priorly histolopathologically diagnosed cases. 500 age and gender matched controls with deleterious habits and without any premalignant lesions were recruited from the Department of oral medicine in Kamineni institute of dental sciences. 

Out of 500 oral cancer patients, a total of 399 (79.8%) were males and 101 (20.2%) were females which are similar to study done by Dhanuthai et al., (2018) where 68.90% were males, 31.07% were females and in a study done by Sherin et al., (2008) oral cancer in young adults showed a 3.5 times higher incidence in males.

In the present study out of 500 cases of carcinoma comparing all 10 subsites of oral cancer, tongue 159 (31.8%) and buccal mucosa 192 (38.4%) combining both left and right side were found to be with higher prevalence and least prevalence was seen with lip 8 (1.6%) which was in accordance with the studies done by Dhanuthai et al., (2018) and Sherin et al., (2008). 

Among 500 oral cancer patients, 147 (29.4%) patients had smoking tobacco and 312 (62.4%) had chewing tobacco and in 500 control group with habits 229 (45.8%) had a smoking habit and 230 (46.0%) had chewing tobacco habit. In a study done by Jain et al., (2003) high prevalence of oral cancer was observed with chewing tobacco (66.2%) than smoking tobacco (33.8%) which is in accordance with the present study. Only 3 patients out of 106 oral cancer with alcohol consumption had no history of any form of tobacco (smoking or chewing), those 3 cases are happened to be on the tongue and due to trauma from the attrited teeth. The other 103 patients had either smoking or chewing tobacco which is in accordance with other studies stating tobacco has a synergistic effect on the development of oral cancer (Agrawal et al., 2003; IARC, 2002).

Diabetes mellitus was confirmed by performing fasting blood glucose levels and postprandial levels in the present study. According to ADA Generally, FPG, 2-h PG during 75-g OGTT, and A1C are equally appropriate for diagnostic testing. Numerous studies have confirmed that compared with FPG and A1C cut points, the 2-h PG value diagnoses more people with diabetes. PPG strongly correlates with HbA1c or contributes significantly to overall glycemic control (Ketema, 2015).

In the present population based study, out of 500 oral cancer patients, 446 (89.2%) were nondiabetic and 54 (10.8%) were diabetic and in 500 controls with habits, 421 (84.2%) were nondiabetic and 79 (15.8%) were diabetic which signifies an inverse association between DM and oral cancer similar to studies done by Amini et al., (2018), Carvalho et al., (2016) but were not in ordinance with few studies such as Boccia et al., (2012); Bíbok et al., (2004) and Gong et al., (2015).

In the present study, out of 54 diabetics in the study group, 39 (7.8%) were metformin users and 15 (3.0%) non-metformin users and out of 79 diabetics in control group 72 (14.4%) were metformin users and 7 (1.4%) were non-metformin users. In comparison, Metformin users in the present study had a lower risk of oral cancer. No significant association was observed with non-metformin users. The possible reason for this could be metformin use may be explained by its role in the activation of adenosine monophosphate-activated protein kinase (AMPK). AMPK inhibits protein synthesis and the action of the mTOR protein. The mTOR pathway is regulated by both AMPK and AKT, which have opposite functions. In response to an external stimulus, AKT activates the mTOR pathway, contributing to cell proliferation. On the other hand, AMPK can reduce the action of mTOR. Metformin use may activate AMPK, thus inhibiting mTOR action and decreasing cell proliferation which was in accordance with some studies (Agarwal etal., 2012; Gander et al., 2014; Lin et al., 2015; Bowker et al., 2006).

In the present study, study group showed 6 (4.1%) of metformin users of which 1 case had alcohol habit and control group showed 42(18.3%) of metformin users of which 6 cases had alcohol habit who were smokers which were statistically significant and alcohol may have synergistic effect which was in accordance with the study done by Carvalho et al., (2016). The reasoning may be Serine/threonine-protein kinase AKT is a downstream target of phosphatidylinositol 3-kinase and a key regulator of normal and cancerous growth and cell fate decisions. Although tobacco and alcohol consumption are the main risk factors for HNC, the molecular mechanism involved is not clear and few studies have investigated the association between these risk factors and AKT and mTOR activation, especially in HNC. One study showed that alcohol and tobacco activate AKT. Higher phosphorylation of Akt at T308 as a reliable biomarker for smoking and alcohol induced HNSCC progression. Another study showed that NKK, the main tobacco carcinogen, is associated with the activation of AKT in HNC patients. AKT is activated at a higher frequency in both HNSCC tumors and the adjacent mucosa from HNSCC patients who are smokers than those from HNSCC patients who are non-smokers. Adding physiologically relevant concentrations of 4-(methylnitrosamino)-1-(3-pyridyl)-1- 1butanone (NNK), a major tobacco carcinogen, to the normal head and neck epithelial cells and HNSCC cell lines, rapidly and constitutively activated AKT through phosphorylation in a dose- and time-dependent manner. AKT phosphorylation was associated with activation of downstream signaling mediators BAD, MDM2, GSK-3β, mTOR. These alterations correlated with increased proliferation and decreased etoposide-induced apoptosis in NNK-exposed cells. Finally, NNK exposure to mouse head and neck epithelia resulted in epithelial hyperproliferation and reduced apoptosis, which is correlated with AKT activation (Chen et al., 2001; Cochrane et al., 2014).

Thus the present population based study results suggest that AKT activation is an early event and plays a pivotal role in mediating tobacco induced oral carcinogenesis and metformin inhibits the AKT activation. This is the first study correlating the association of DM and oral cancer with metformin and first of its kind in assessing the role of habits in association with DM and Metformin in the etiology of oral cancer.
